# Realtime Tracking of Passengers on the London Underground Transport by Matching Smartphone Accelerometer Footprints

**DOI:** 10.3390/s19194184

**Published:** 2019-09-26

**Authors:** Khuong An Nguyen, You Wang, Guang Li, Zhiyuan Luo, Chris Watkins

**Affiliations:** 1 Computer Learning Research Centre, Computer Science Department, Royal Holloway University of London, Surrey TW20 0EX, UK; 2 State Key Laboratory of Industrial Control Technology, Institute of Cyber-Systems and Control, Zhejiang University, Hangzhou 310027, China

**Keywords:** underground transport tracking, inertial dead reckoning, sensor trace matching

## Abstract

Passengers travelling on the London underground tubes currently have no means of knowing their whereabouts between stations. The challenge for providing such service is that the London underground tunnels have no GPS, Wi-Fi, Bluetooth, or any kind of terrestrial signals to leverage. This paper presents a novel yet practical idea to track passengers in realtime using the smartphone accelerometer and a training database of the entire London underground network. Our rationales are that London tubes are self-driving transports with predictable accelerations, decelerations, and travelling time and that they always travel on the same fixed rail lines between stations with distinctive bumps and vibrations, which permit us to generate an accelerometer map of the tubes’ movements on each line. Given the passenger’s accelerometer data, we identify in realtime what line they are travelling on and what station they depart from, using a pattern-matching algorithm, with an accuracy of up to about 90% when the sampling length is equivalent to at least 3 station stops. We incorporate Principal Component Analysis to perform inertial tracking of passengers’ positions along the line when trains break away from scheduled movements during rush hours. Our proposal was painstakingly assessed on the entire London underground, covering approximately 940 km of travelling distance, spanning across 381 stations on 11 different lines.

## 1. Introduction

Everyday, over 3.75 million passengers use the London underground tube, with an average of 20 min on board per person (https://tfl.gov.uk/corporate/about-tfl/what-we-do/london-underground/facts-and-figures—last accessed in May 2019.). However, since the London underground network is nearly 60 m below street level, satellite and terrestrial signals cannot penetrate the ground, creating a black zone where signal-based services such as Google Maps cannot operate. Two existing solutions attempted by Transport for London (TfL) only indicate the beginning and the end of the passengers’ trip by reading when they tap their Oyster card at each station or by using the Wi-Fi Access Points (APs) installed at each station platform to detect the passengers’ presence. The challenge for both approaches is that they cannot monitor the passengers in realtime on the lines, as there are no Wi-Fi APs on the train or inside the tunnels. As such, there is a demand for a ubiquitous technology to deliver live tracking for passengers on the underground trains.

The reason a realtime underground tracking system is desirable is because of high practical value for tourists, commuters, and line managers. For first-time visitors, our system informs them of their whereabouts, since it is easy to mistakenly board the wrong train travelling in the opposite direction on the same line. Daily commuters will be able to estimate how far away the destination is, which is especially valuable during rush-hour delays, without having to rely on public announcements. For the transport managers, such system provides a means of congestion planning (i.e., knowing which parts of the line, which stations, are most busy during certain periods) and epidemic tracking (i.e., identifying potential disease contact of people travelling on the same route at the same time).

In this article, we present a novel approach to track underground passengers in realtime, with only the smartphone accelerometer and a training database recording the predetermined train’s acceleration, the trains’ deceleration, and the shape of the tunnels. We applied several filters to get rid of the noises generated by human gestures and to expose the true train movements. Overall, the key advantages of our work are as follows:

- First and foremost, the whole system requires no additional infrastructure or hardware to be installed anywhere on the underground network. It leverages the user’s smartphone to provide the tracking service.
- The system is self-contained in the form of an app. The inputs will be recorded by the app, which also contains the training database and the route estimation mechanism.
- Only low-power accelerometers with minimal impact on battery consumption is used.
- Although privacy is not our primary focus, it is worth adding that no internet connection is needed for our system. The positioning service is delivered completely offline and locally via the app. Hence, the user may remain anonymous while using the service, if so wished.

The remainder of the article is organised into eight sections. Section 2 tells the story behind our idea of using the smartphone accelerometer to track passengers undergrounds, with an emphasis on the challenges facing such an approach. Section 3 then explains the first steps in compiling our training database so that Section 4 can build on it to match the realtime traces. Section 5 addresses the challenging scenario where a train stops midway. Section 6 and Section 7 detail the empirical results. Section 8 overviews the related work. Lastly, Section 9 summaries our work and outlines potential expansion.

## 2. Underground Transport Tracking with Smartphone Accelerometer

**Figure 1:**
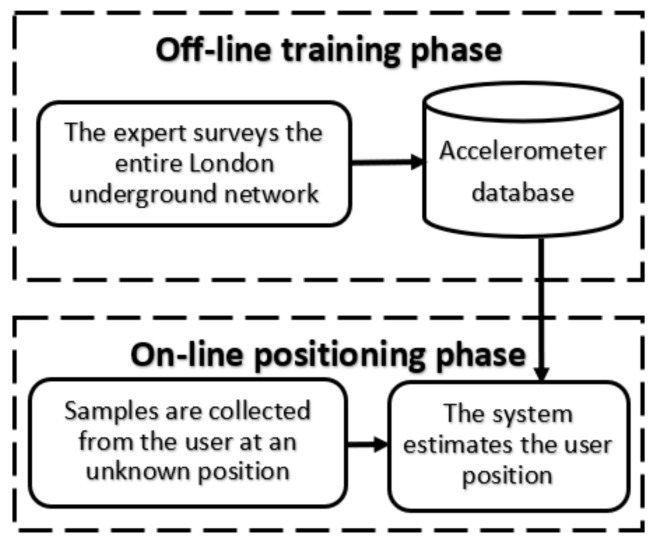
The two phases of our underground tracking system.

This section introduces our motivation for using the accelerometer to track underground passengers and the challenges facing such an approach. In essence, our system has two phases (see Figure 1). In the offline phase, we generate a training database surveying the train’s movements throughout the entire underground network. In the online phase, the passenger’s inputs will be matched against our database. Each phase will be investigated in detail in the upcoming sections.

### 2.1. The Inspirations of Using Accelerometer for Passenger Tracking

The rationales of our approach stemmed from many hours of observing the physical map of the London underground and commuting on the tube itself daily. They can be summarised in four points.

**Figure 2:**
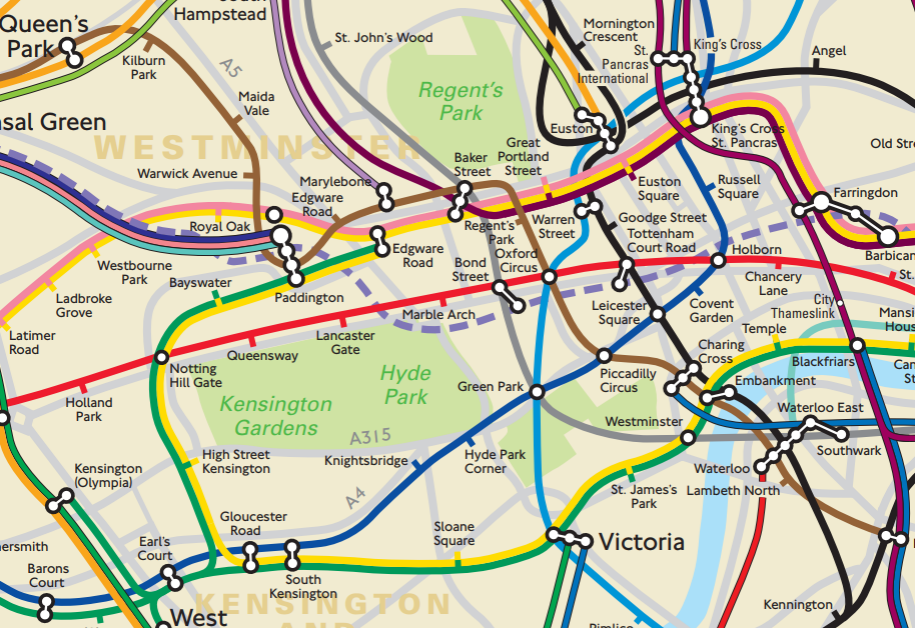
A part of the actual physical underground network in central London embedded on a map: The distance between consecutive stations is short.

- **The rail-line infrastructure is fixed amongst stations** All trains must go through the same predetermined route connecting 2 consecutive stations. Therefore, it is possible to record the shape of the underground infrastructure in terms of the sensor language into a training database. As the London underground network is over 150 years old, their structure does not change significantly over time, making it much easier to maintain such a database.
- **London underground tubes are autonomous vehicles.** Despite not being advertised widely, London tubes are programmed to operate automatically. This means the states of acceleration from a station, of deceleration until the destination, and of running between stations are reproducible and may be matched against the above training database. More importantly, there are currently 8 different rolling stocks operating on 11 London underground lines. Each of them accelerates and decelerates differently.
- **The trip between two consecutive underground stations is short** (see Figure 2). This characteristic is important to control the nature of sensor drifting, which will happen over time as error quickly accumulates.
- **Underground carriages have limited space while running at high speed.** Being in a confined environment would limit passengers’ movements. They would normally have to sit down or hang onto the handrails and hanging straps while standing still, which enables the phone to measure the true movements of the train (http://content.tfl.gov.uk/london-connections-map.pdf—last accessed in May 2019).

### 2.2. Mobile Accelerometer

**Figure 3:**
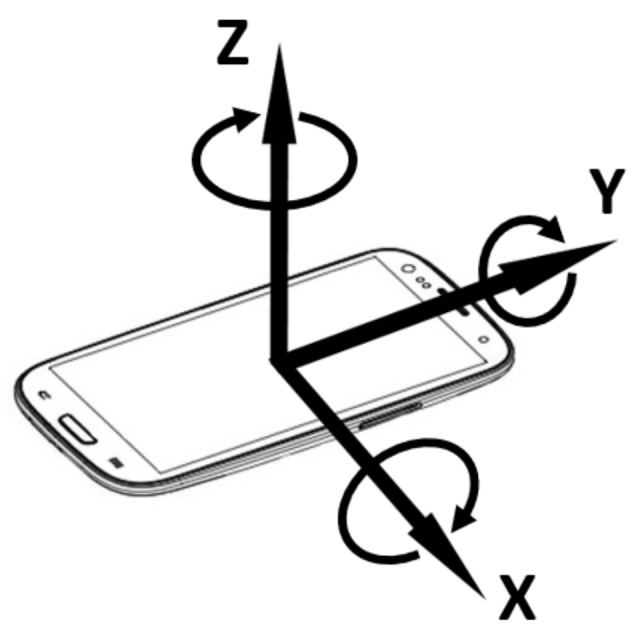
The three measures returned by the accelerometer correspond to the orientation of the smartphone in three dimensional space.

Concisely speaking, the smartphone accelerometer is an electromechanical sensor used to measure acceleration forces (i.e., the changing rate of the velocity or speed divided by time) along the three Cartesian axes at any given time (see Figure 3). Such forces can be static (e.g., continuous gravitational pull) or dynamic (e.g., vibrations and movements). The measuring unit is in m per second squared (m/s${}^{2}$).

In 2019, the accelerometer is ubiquitous in every smartphone to serve the most basic function of detecting the phone’s orientation (i.e., portrait or landscape) for the user interface. The accelerometer is part of a group of so-called low-power sensors (along with the gyroscope, magnetometer, and barometer), which have the distinctive features of consuming modest power, being always-on, and requiring no permission from the user to operate.

### 2.3. The Challenges

Despite the aforementioned advantages, do bear in mind that the sensor is designed with affordability, power efficiency, and simplicity in mind rather than purposely for our underground tracking system. Therefore, the following challenges should be addressed.

- **Unconstrained sensor orientation**. The accelerometer’s coordinates align with the phone’s 3-dimensional frame. As such, the measures vary as the phone’s orientation changes, despite being in the same position. The challenge is we do not know how the passenger is holding the phone. The magnetometer, often used to estimate true north, is unfortunately not usable in our case, as magnetic disturbance is too high underground.
- **Unrelated measures**. Small human gestures (e.g., leg shaking and swift arm moving) and electrical noises may be mistakenly as train movements.
- **Unexpected train movements**. During rush hours, trains may not adhere to scheduled movements by stopping midway due to congestion, which invalidates the use of our training database.

A combination of these factors may obfuscate the real acceleration and deceleration of the train and the current passenger position that we would like to inspect. In the upcoming sections, we will introduce our techniques to address these challenges.

## 3. Compiling the Training Database

This section presents the process to generate our unique accelerometer training database, which records the tube’s movements on all 11 London underground lines in both directions (see Table 1). In essence, we aim to collect pure train-related data, free of external noises, with decisions taken to ease the sample labelling process later on. As such, the following actions were taken.

- **The surveyor’s phone was rigidly attached to the seat.** Throughout the surveillance, the phone was fixed to the train seat with tapes to avoid any external movements apart from that of the train.
- **The phone accelerometer’s*****Y*****-axis was aligned to the train’s heading.** By positioning the phone flat in parallel with the carriage floor and by pointing to the moving direction, we enable the train movements to concentrate on the *Y*-axis and to leave the gravitational force on the *Z*-axis of the accelerometer.
- **The accelerometer samples were labelled manually.** As there is no other independent positioning system that works underground, we will manually examine the acceleration and decleration to convert them into velocity, which in turns reports the travelling distance from the last station, to be used as a label. Each sample is also accompanied by a time-stamp and labelled with the line and the station stop accordingly.
- **Assessment time was late morning.** This is the super off-peak hours where London trains run smoothly, without any incidents or delays.
- **The database is fine-grained.** Ideally, the accelerometer’s sampling rate should be as high as possible to record the slightest change in motion. However, the higher the sampling rate is, the bigger the training data, the higher the energy consumption, and the slower the positioning estimation will be. One of the first works in this domain tried to classify physical human activities that exhibit frequent changes of motion (e.g., walking, running, ascending stairs, etc.), which concluded that a 15–20 Hz was sufficient, with virtually no accuracy improvement with higher sampling rates [1]. Subsequently, most recent works in transportation mode detections with a smartphone accelerometer achieved high estimation accuracy with 35 Hz, 32 Hz, and 16 Hz respectively [2,3,4]. As such, we settled on a relatively safe, above-literature threshold of 50 Hz (50 samples per second) to generate our training database.

In total, we recorded 4,479,900 accelerometer samples on 740 single routes over 381 stations in both directions. The entire database is about 150 MB. A detailed breakdown of each line is summarised in Table 1. The whole training process took almost 25 h, which is purely train riding time, without the preparation, setup, and change between trains factored in.

### Labelling the Training Samples with Estimated Travelling Distance

Since there is no other independent tracking system to provide a positioning label for each accelerometer sample, we will use the accelerometer reading to estimate the travelling distance from the last station stop. This process strongly benefits from our planned actions taken while recording the database, to be explained below.

Given the accelerometer trace between two consecutive station stops with *N* samples $\left(\vec{t_{1}},\cdots ,\vec{t_{N}}\right)$, where each $\vec{t_{i}}=\left(a_{x}^{i},a_{y}^{i},a_{z}^{i}\right),1\leq i\leq N,$ is the training sample vector reported by the smartphone’s accelerometer and where $a_{x},a_{y},a_{z}$ are the acceleration measured along each of the three axes, each sample’s velocity and distance are calculated as follows.

(1) $$v\left(i\right)=v\left(i-1\right)+a_{y}^{i}*t$$

(2) d(i)=d(i−1)+v(i)∗t

where v(0)=0 and d(0)=0 are the initial velocity and distance, respectively, and *t* is the regular sampling interval (e.g., 20 ms). Note that we are only interested in $a_{y}$ because the phone’s *Y*-axis was aligned to the train’s heading, which helpfully leaves out the gravitational force to the *Z*-axis.

Lastly, the label of each training sample is a tuple composed of the distance label computed by Equation (2), the corresponding station stops, the underground line, as well as a time-stamp of when the measurement happened.

## 4. Matching Passengers’ Accelerometer Footprints

Having discussed the idea behind our training database, we are now in a good position to explain the process of collecting the passenger’s realtime data and to match them against our training samples.

### 4.1. The Android App

**Figure 4:**
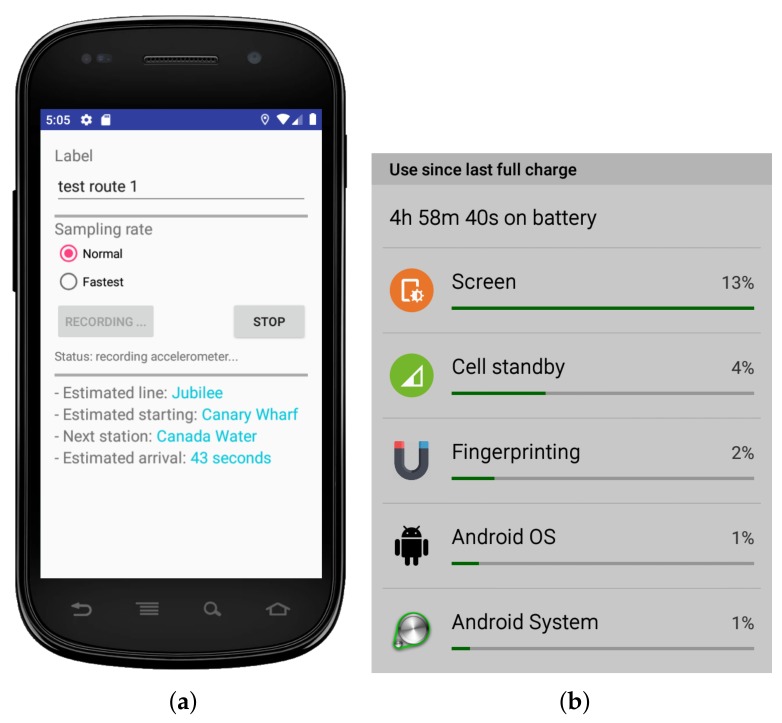
The Android app used to collect accelerometer data on the passenger’s phone: (**a**) The app interface and (**b**) The power consumption.

For the purpose of this work, we developed a proof-of-concept Android app to be installed on the passenger’s device (see Figure 4a). The app serves three purposes.

- It stores the above training database.
- It silently collects the accelerometer data in the background, while freeing the user to carry on with daily activities.
- It performs realtime matching of passenger data to estimate the travelling route to be discussed below.

The sensor’s measures will be saved locally on the phone but only temporarily for the realtime matching purpose. As soon as a matching is found or when the app is closed, these measures are erased for security reasons. No internet connection is needed at any time.

For completeness, we report the power consumption of our Android app. Figure 4b demonstrates that the app consumes as little as 2% of the battery per charging cycle, despite being in use constantly for nearly 5 h. It is worth pointing out that our current procedure could be further optimised by reducing the accelerometer’s sampling rate and by increasing the trace’s step length to speed up the inquiring process.

### 4.2. Handling Unconstrained Phone Placements

**Figure 5:**
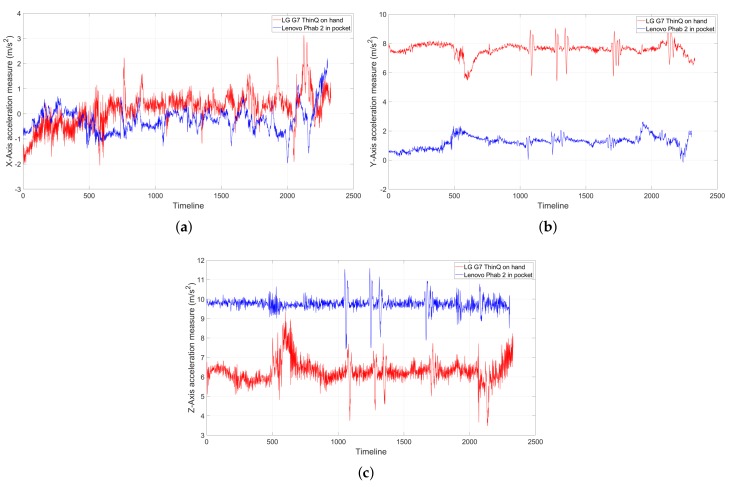
The accelerometer measures of individual axes of two phones in the same position yet with different placements over the same trip: Their measures may vary significantly. (**a**) *X*-axis; (**b**) *Y*-axis; and (**c**) *Z*-axis.

The challenge with processing the passenger’s data is that we do neither know how they hold the phone nor can we enforce a fixed orientation of the device through out the journey. The consequence is that, even in the same position, different orientations vary the accelerometer’s (x, y, z) measures to an extent that is completely different from the original samples in the training database for matching (see Figure 5). In our circumstance, we are mostly limited to just the accelerometer measures, since the magnetic perturbance is too great underground to discover true north with the magnetometer, as often used in outdoor, overground navigation.

The simplest and most robust solution is to take the total scalar magnitude for every accelerometer sample vector $\vec{t}=\left(a_{x},a_{y},a_{z}\right)$ as follows. This approach is sufficient for our matching mechanism when we only consider the change of acceleration over time.

(3) $$\left|\right|\vec{t}\left|\right|=\sqrt{a_{x}^{2}+a_{y}^{2}+a_{z}^{2}}$$

Nevertheless, with this approach, we lose the the direction of movement in the process, and thus, cannot determine if the force is accelerating or decelerating, which is of particular importance to our purpose to compute the velocity later on with inertial tracking in Section 5.

As such, we implement a method to rotate the passenger’s arbitrary accelerometer vector to the same coordinate system correlated to gravity [5,6]. This approach exploits the fact that the accelerometer is under the influence of the gravitational force at all times, beside the train movements. Also, we know that gravity is always pulling the device towards the centre of the Earth in the same direction, with the same force measured at 9.8 m/s${}^{2}$. The algorithm works as follows.

Given a segment of *K* continuous accelerometer measures $\left(\vec{t_{1}},\cdots ,\vec{t_{K}}\right)$, where $\vec{t_{i}}=\left(a_{x}^{i},a_{y}^{i},a_{z}^{i}\right),1\leq i\leq K$, the estimated gravity vector $\vec{v}=\left(v_{x},v_{y},v_{z}\right)$ for this period is computed by averaging the measures on each axis as follows. The rationale is that, although $\left(a_{x},a_{y},a_{z}\right)$ contains other forces beside gravity, by averaging the measures, we bring them to a constant value. A constant acceleration means the device is not moving at all.

(4) $$\vec{v}=\left(\frac{\sum_{i=1}^{K}a_{x}^{i}}{K},\frac{\sum_{i=1}^{K}a_{y}^{i}}{K},\frac{\sum_{i=1}^{K}a_{z}^{i}}{K}\right)$$

Then, we compute the true dynamic motion for each measure within the above sampling period by subtracting the gravitational vector as follows.

(5) $$\vec{d_{i}}=\left(a_{x}^{i}-v_{x},a_{y}^{i}-v_{y},a_{z}^{i}-v_{z}\right)$$

Then, we project this new vector onto the gravity vector as follows, where $\vec{p_{i}}$ is the vertical component of $\vec{d_{i}}$ as a result.

(6) $$\vec{p_{i}}=\frac{\vec{d_{i}}\cdot \vec{v}}{\left|\right|\vec{v}{\left|\right|}^{2}}\vec{v}$$

Then, we compute the horizontal component $\vec{h_{i}}=\left(h_{x}^{i},h_{y}^{i},h_{z}^{i}\right)$, which is perpendicular to $\vec{d_{i}}$ as follows.

(7) $$\vec{h_{i}}=\vec{d_{i}}-\vec{p_{i}}$$

As the underground tunnel is relatively flat, the above horizontal vector $\vec{h_{i}}$ should lie on the orthogonal plane to gravity. Since, we have subtracted the gravitational force from each accelerometer measure, we may ignore the third *z*-gravity component and maintain just a single data point on this plane.

**Figure 6:**
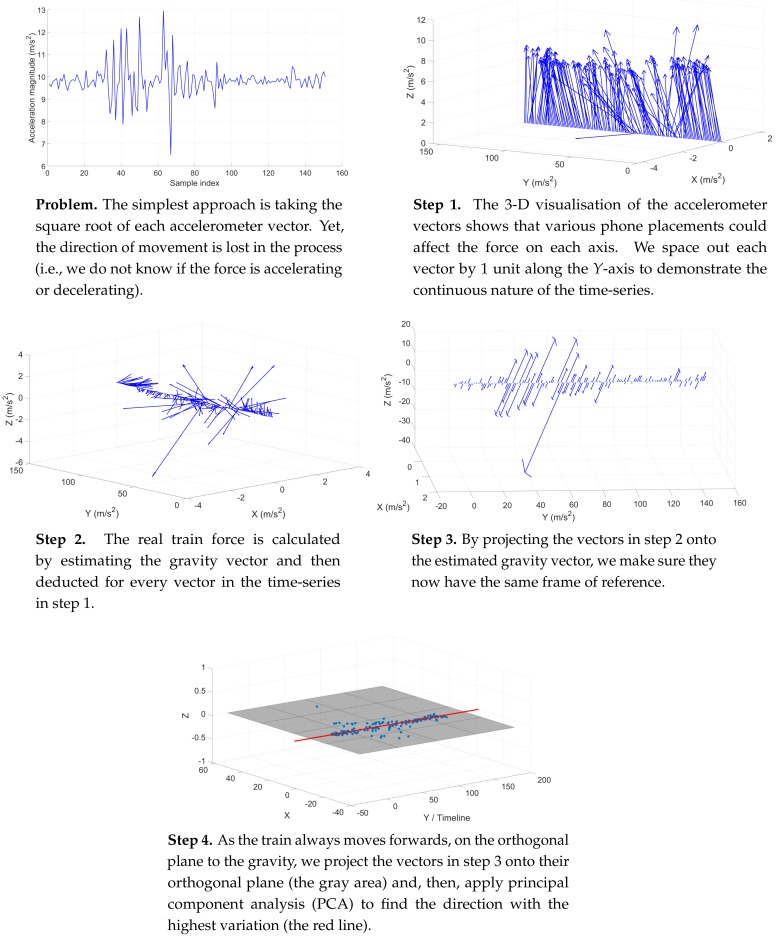
A visual demonstration of the step process to handle unconstrained phone placements and to obtain real train accelerations using PCA on a real-world time-series of 150 accelerometer measures.

To estimate the true train motion, we compute the above horizontal vector $\vec{h_{i}}$ for all accelerometer samples within a continuous time window. Since the train only moves forward throughout a trip, we assume that all accelerations and decelerations happen in one direction. As such, by applying Principal Component Analysis (PCA) on the above *K* data points, we may estimate the train force as the first PCA component, the one with the most variation (see Figure 6), with the following steps [7,8].

We first subtract the mean from each data dimension for every data point.

(8) $$\vec{h_{i}^{\prime }}=\left(h_{x}^{i}-\bar{h_{x}},h_{y}^{i}-\bar{h_{y}}\right)$$

Then, we compute the following covariance matrix.

$$A=\left(\begin{array}{cc} \sigma (h_{x}^{\prime },h_{x}^{\prime }) & \sigma (h_{x}^{\prime },h_{y}^{\prime })\\ \sigma (h_{y}^{\prime },h_{x}^{\prime }) & \sigma (h_{y}^{\prime },h_{y}^{\prime }) \end{array}\right)$$

where $\sigma \left(h_{x}^{\prime },h_{y}^{\prime }\right)=\frac{1}{K-1}\sum_{i=1}^{K}$*h*’_*x*_^*i*^ *h*’_*y*_^*i*^

We then compute the eigenvectors and the corresponding eigenvalues of the above covariance matrix and sort them by eigenvalue from the highest to the lowest. The result is the PCA components in their order of significance. For our purpose, we pick the first (i.e., the most significant component). Lastly, we convert each accelerometer measure in the sampling period into a new estimated measure as follows. First, we transpose the above eigeinvectors so that their rows are now the eigenvectors, with the most significant eigenvector at the top. Then, we transpose the mean-adjusted data so that the data items are in each column and each row represents a separate dimension.

### 4.3. Filtering the Noises

Despite being confined in a limited space and having to sit down or to hang onto the handrails while the tube is moving at high speed, some occasional human movements may be recorded. Additionally, electrical noises may pollute the accelerometer measures. This section will address those issues accordingly.

**Figure 7:**
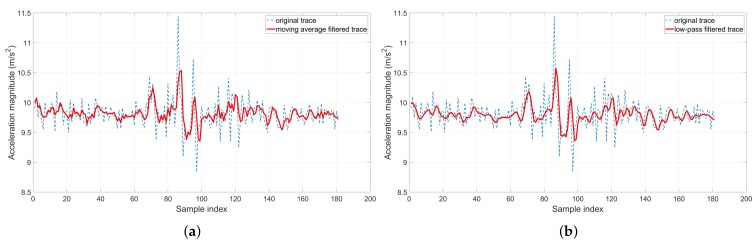
A demonstration of the moving average filter and low-pass filter in action: They significantly reduce the sensor noises and deliver smoother traces. (**a**) Moving average filter; (**b**) Low-pass filter.

Firstly, to filter out the short-term electric noises, we apply a linear moving average filter on the passenger’s samples (see Figure 7a). An empirical window size of 10 samples was applied, as there are up to 50 samples per second. Without loss of generality, given the accelerometer trace of a route $\left(a_{1},\cdots ,a_{N}\right)\left(11\leq i\leq N\right)$, where $a_{i}$ is either the total scalar magnitude or the estimated acceleration measure obtained in the last section and *N* is the length of the trace, each sample will be smoothed out as follows.

(9) $$a_{i}^{\prime }=\frac{\sum_{j=1}^{10}a_{i-j}}{10}$$

Secondly, to address the human noises, we observe that London trains do not stop or increase speed abruptly, whereas swift human gestures tend to cause a short, sudden peak in the accelerometer measures. However, the above moving average filter tends to respond slowly to the quick changes in time-series traces. Therefore, we apply a low-pass filter to smooth out these anomalies (see Figure 7b). More importantly, trains only move forward in one direction. In contrast, the passenger’s hand and leg gestures normally follow up by the same gesture in the opposite direction. Hence, speed-wise, these gestures cancel themselves out.

(10) $$a_{i}^{\prime }=a_{i-1}^{\prime }+\alpha \times \left(a_{i}-a_{i-1}^{\prime }\right)$$

where α is the positive smoothing factor, (2≤i≤N). A smaller α makes the filter respond slowly but is more robust to noises, whereas a bigger one responds faster but is more sensitive to noises. An empirical α=0.35 was found to deliver the best performance in our experiments.

### 4.4. Extracting the Train Traces from the Passenger’s Sensor Recording

To extract only the relevant train-related accelerometer measures, we need to determine the starting point when the passenger gets on the train and the cut-off point when they get off the train within the long continuous time-series of sensor recordings. This problem becomes more challenging since they may spend time walking through the platforms of different lengths before getting on the train. To assist this process, we define three states of motion for each sensor sample.

**Figure 8:**
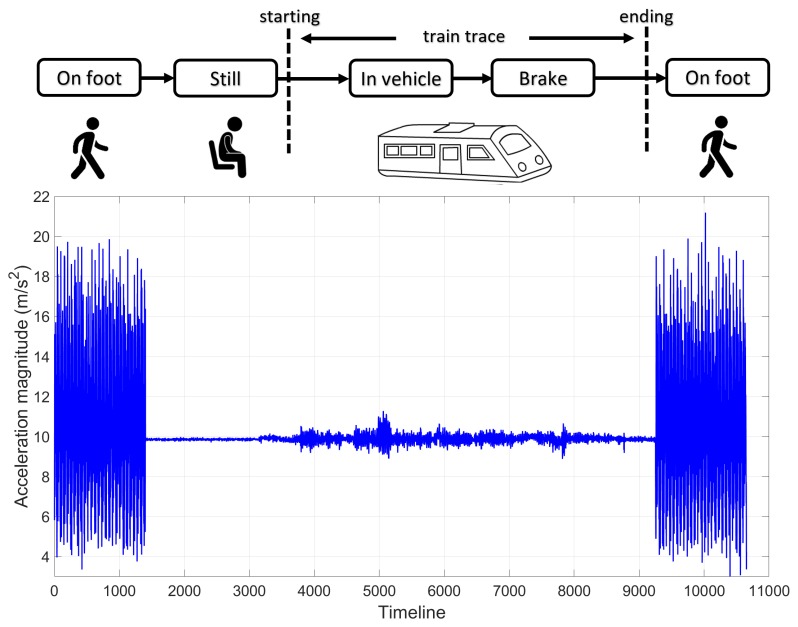
The activity recognition process to determine the starting and ending points of a train trace.

- **On foot state**. This motion corresponds to the passenger walking from the station concourse onto the train and vice-versa. It has the distinctive shape as visualised in Figure 8.
- **Still state**. This is the state where the passenger is motionless and is easily detected when no acceleration (minus the constant gravity force) is reported by the accelerometer.
- **In-vehicle state**. This motion is triggered when the passenger is on the train and is detected by the fast accelerations at the beginning of the trip.

To identify the above motion states, we employ the built-in Android Activity Recognition API (https://developers.google.com/android/reference/com/google/android/gms/location/ActivityRecognitionClient—last accessed in May 2019). This engine is based on the Bayesian classifier, with a training set of 6 different sensor motions (walking, running, still, on foot, on bicycle, and in vehicle). We will only be interested in the “on foot” and “in vehicle” activities. To avoid the false recognitions, we will take the majority of the activities over a time window.

Based on the above motion states, we make the following observations of how the sensor sample moves from one state to another to determine the starting and ending of a train trace (see Figure 8).

- **Starting point**. The passenger must walk from the station platform onto the train. This is followed by a brief period of inactivity and ends with the train moving forwards.
- **Ending point**. London underground tubes always brake to a swift stop by the end of the trip. This motion is followed by the passenger walking off the train.

### 4.5. Finding the Single Matching Training Route

The above passenger’s trace after being processed will be matched against all training routes to find the most similar one. To determine the similarity of two accelerometer traces, we employed Derivative Dynamic Time Warping (DDTW) [9,10] for four reasons.

**Figure 9:**
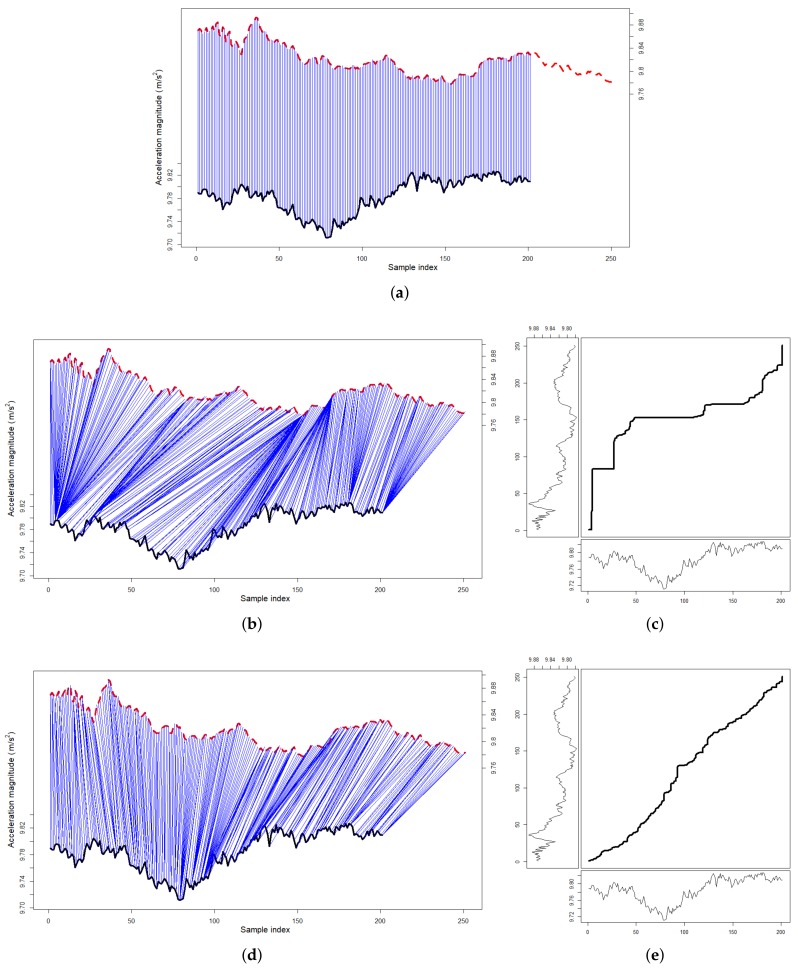
The justification of using Derivative Dynamic Time Warping (DDTW) to match accelerometer traces: Euclidean distance-based matching fails to align traces of various lengths. Standard Dynamic Time Warping (DTW) overwarps the *X*-axis when the variability on the *Y*-axis is high. (**a**) Euclidean alignment; (**b**) DTW alignment; (**c**) DTW warping path; (**d**) derivative DTW alignment; and (**e**) derivative DTW warping path.

Firstly, Dynamic Time Warping (DTW) is capable of matching misaligned traces caused by various sensitivities from different phone accelerometers by looking for an optimal warping path, whereas other distance-based approaches (e.g., Euclidean and Manhattan) simply align the *i*th point on one trace to the same *i*th point on the other trace (see Figure 9a). Secondly, it can stretch the shorter trace with a lower sensor sampling rate to match our long training traces. Thirdly, DTW is a proven algorithm with well-known speech recognition research applications [11,12,13,14,15]. Fourthly, while standard DTW may overwrap the *X*-axis by mapping one single sample on the passenger’s trace onto multiple samples on the training’s trace, if the variability of the *Y*-axis is large, DDTW overcomes this challenge (see Figure 9b,d).

Without loss of generality, given two accelerometer time-series traces $A=(a_{1},\cdots ,a_{m})$ and $B=(b_{1},\cdots ,b_{n})$, where $a_{i},b_{i}$ are the scalar magnitudes of the accelerometer readings, we first perform Z-normalisation on the two time-series [16]. This preprocess step makes sure that the measures have an approximate zero mean and that the standard deviation is in a range close to 1, which were reported to benefit DDTW later on [17,18,19]. Then, we transform the original points into higher-level features to expose the shape-related information of the time-series and to eliminate any variability as follows [9].

(11) $$a_{i}^{\prime }=\frac{a_{i}-a_{i-1}+\left(\left(a_{i+1}-a_{i-1}\right)/2\right)}{2},\left(1<i<m\right)$$

(12) $$b_{j}^{\prime }=\frac{b_{j}-b_{j-1}+\left(\left(b_{j+1}-b_{j-1}\right)/2\right)}{2},\left(1<j<n\right)$$

As the first and last points are not defined, we consider $a_{1}^{\prime }=a_{2}^{\prime },a_{m}^{\prime }=a_{m-1}^{\prime }$ and $b_{1}^{\prime }=b_{2}^{\prime },b_{m}^{\prime }=b_{m-1}^{\prime }$.

Then, we construct an m-by-n matrix, in which each [i,j] cell contains the distance $d(a_{i}^{\prime },b_{j}^{\prime })$ calculated as follows.

(13) $$d\left(a_{i}^{\prime },b_{j}^{\prime }\right)={\left(a_{i}^{\prime }-b_{j}^{\prime }\right)}^{2}$$

Next, we find the optimal contiguous warping path of length *k*: w(1,1),⋯,w(m,n) between the two traces that minimises the warping cost $\sum_{i=1}^{k}w_{i}$ by using the following recursive step.

(14) $$w\left(m,n\right)=d\left(a_{i}^{\prime },b_{j}^{\prime }\right)+min\left\{\;\begin{array}{c} w(i-1,j)\\ w(i-1,j-1)\\ w(i,j-1) \end{array}\;\right\}\;$$

with (1<i<m,1<j<n).

Since the routes have various lengths, which may return different warping paths, we normalise the DDTW score by the length of the found optimal warped path. The training route with the smallest score will be chosen as the resulting route.

### 4.6. Matching Continuous Trip

If the passenger stays on the train beyond the next station stop (i.e., the trace contains multiple stops), we take advantage of the fixed route length statistics (i.e., trains run for the same amount of time between two consecutive stations) by filtering out the training routes of which the first segment’s length does not match. As we will see in Section 7.6, this information is very helpful to enhance the route estimation accuracy.

**Figure 10:**
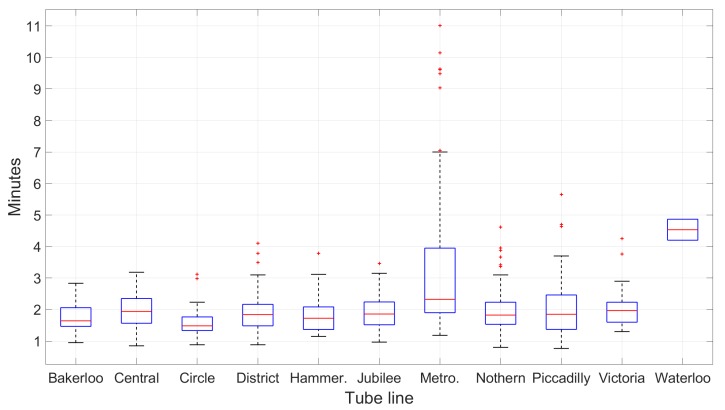
Overview of the travelling time between consecutive stations on all 11 lines: The majority of one-stop journeys fall between 1 min and 3 min. For each box, the red central line indicates the median; the bottom and top edges indicate the 25th and 75th percentiles. The whiskers extend to the furthest non-outlier data points, while the outliers are plotted individually as “+”.

Broadly speaking, the majority of one-stop journeys across all lines falls between 1 min and 3 min, with some exceptionally long routes of up to 11 min. However, looking closely at continuous journey on each individual line reveals that the amount of time to travel between consecutive stations vary greatly (see Figure 10). This property is important for matching long journeys containing multiple stops.

### 4.7. Validating the Matching Route

The challenge for our DDTW matching, in particular, and other distance based matchings, in general, is that the algorithm will always return a best-matched training sample (i.e., the training route with the smallest difference measure to the passenger trace) despite the reality that both traces may not be similar at all. Therefore, we present the following heuristics for the system to judge whether the returned route is indeed similar.

- **The passenger trace length is equivalent to at least 1 stop.** This empirical threshold ensures that the sensor trace is distinctive enough and avoids too many similar false matchings; this will be evaluated in Section 7.6.
- **The DDTW score is below an empirical constant of 0.3**. This score is calculated by adding up the differences between all aligned samples on the accelerometer trace, divided by the length of the optimal warped path; this will be evaluated in Section 7.5.

Any pair must satisfy the above criteria to be considered a valid match. We will evaluate their performances in the experimental section.

However, the challenge for our matching mechanism discussed so far is that our training database cannot be used when trains do not follow scheduled movements (e.g., unplanned mid-route stops due to congestion during rush hours). The next section will discuss our implementation of real-time positioning estimations in such scenarios.

## 5. Estimating the Passenger Position with Inertial Tracking

Having explained the concept of accelerometer trace matching, we are now in a good position to address the challenging scenario, where there is no matching trace due to the train stopping midway or where the train does not follow the schedule during rush hours. It is worth emphasising that this inertial tracking module will only engage when we already know which line the passenger is travelling on and which station the passenger departs from, provided by the above pattern matching mechanism. There is little meaning in monitoring how far the passenger has travelled if we do not know their initial position or where the destination is. As such, this mechanism is only triggered when three conditions are met. Firstly, the above matching engine must already find a matching route in the training database. Secondly, the passenger has not reached the destination. Thirdly, the current best matched route fails the validation process, as outlined in Section 4.7.

**Figure 11:**
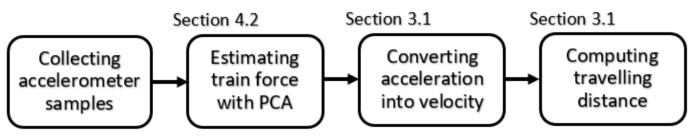
The step process of estimating the travelling distance given the passenger’s accelerometer measure.

The mechanism works as follows. First, we estimate the true train acceleration with PCA, as explained earlier in Section 4.2. Then, we convert this measure into the accumulated velocity and the estimated travelling distance in the same manner as labelling the training samples, as explained in Section 3.1. Without having to repeat all the equations, we refer the readers to the above sections and only outline the step process here (see Figure 11).

Nevertheless, it is widely anticipated that most dead reckoning approaches have a high degree of error because of the sensor’s integral drifting. Any small error in the acceleration estimation will translate into larger errors in the velocity and position estimations. The rationale for our approach is that the trip between consecutive stations is short and that passengers are confined in train carriages with limited space. These factors help to control the nature of sensor drifting, to be evaluated in Section 7.

## 6. Bringing It All Together

**Figure 12:**
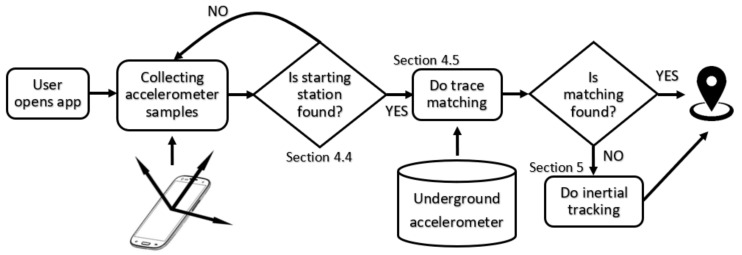
The decision process of doing real-time trace matching and inertial tracking.

Having explained our concept of inertial tracking and sensor footprints matching, each with their own roles, we are now in a good position to combine them into one unified system to deliver real-time passenger tracking (see Figure 12).

When the user opens the app and starts recording, it will collect the accelerometer samples and will attempt to identify the beginning of the train trace. When the starting station is found, the system looks through the training database to find a best match. If the matching engine fails midway, the inertial tracking engine will kick in.

## 7. Experimental Results

This section conducts comprehensive experiments to assess the feasibility and accuracy of our approach by examining every single possible trip on the London underground produced by 4 test phones over a 5 month period. In doing so, it aims to address the following research questions that are most interest to our readers.

### 7.1. Research Questions

In terms of feasibility,

- **How much temporal variation of the accelerometer trace is on the same route?** Ideally, we should observe a similar trace whenever the train travels on the same route because the transport is autonomous.
- **How distinguishable are the accelerometer footprints on all routes?** This question is useful in finding which route the passenger has been or is travelling on without being explicitly told by the user. Since trains accelerate and decelerate differently on each line and the underground tunnels have unique bumps and curves, we surmise that each route has a different accelerometer signature.

In terms of accuracy,

- **How fast and accurate is the rail line positioning?** How quickly and how accurately can the system pinpoint the rail line that the passenger is travelling on amongst 11 different lines with our matching engine.
- **How accurate is the passenger positioning estimation along the line?** As the accelemerometer drifts over time, it is interesting to evaluate the positioning result with our inertial tracking mechanism.

### 7.2. Validation of Results

The challenge to validate the precise positioning and velocity estimation of our approach is that there is currently no accessible underground train positioning system to compare with (although we can easily validate which line and station the user is travelling on). Besides, it is difficult to get the ground-truth speed or location of the train as it travels along the track. Idealistically, the speedometer or tachometer on the train will provide us with the needed ground-truth information. However, getting access to the driver’s cabin was not granted for security reasons. Parts of London underground network are exposed on the surface (e.g. the 3 stations going towards Stratford on the Jubilee line), which allows capturing of GPS signals to work out the true moving speed of the tube on these parts. However, GPS itself has a 10–15 metre positioning error in such conditions and does not provide accurate enough references for our purpose. Hence, we use the following two references to validate our results.

- **Stopping reference**. Trains always stop at each station, which allows us to verify if the estimated speed by the system is indeed zero. In particular, we have 381 such references.
- **Landmark reference**. Some parts of the tunnels are exposed to open air. The interchanges when the train goes in/out of the tunnel can be identified on Google Maps, which provides us the ground-truth distance to the next station. In particular, we manually observed 38 such landmarks (see Table 2).

Nevertheless, it is worth pointing out that these references were visually recorded by the inspector, by manually alerting the Android app when the train stops, and when seeing the landmark as the train passed by. Hence, there would be a certain degree of human error (e.g., the button press happens after the events). With the train travelling at high speeds, we would expect a few m error of tolerance.

### 7.3. Test Devices

Five smartphones were involved in these experiments, namely the LG G7 ThinQ, Samsung J3, Google Nexus 5, Samsung Nexus 2, and Lenovo Phab 2. They were strategically selected to cover a variety of phone models and chip vendors, spread across a number of years (see Table 3). The latest LG G7 ThinQ was used to compile the training database, whereas the other four were used to generate the test data, to be discussed in the next section. Note that we deliberately picked different sampling rates ranging from 10 Hz to 40 Hz for the test phones to reflect the variety of accelerometer capabilities, as reported by a crowd-sourced campaign surveying over 10,000 phones (http://accfreq.cochibo.com—last accessed in May 2019).

### 7.4. Test Data

To assess the feasibility and accuracy of our system, we recollect the accelerometer measures across the entire London underground network, with the 4 test devices (Samsung J3, Lenovo Phab 2, Google Nexus 5, and Samsung Nexus 2) in the same manner as when compiling the training database (outlined in Section 3). However, there are two distinct differences this time. Firstly, the test phones were kept in different body places (e.g., in a bag, in a trouser pocket, in a shirt pocket, and held at various orientations in the hand). Secondly, we generated the test data over two instances (see Table 4).

In the first instance, the test data were measured at different times of the day, when there is no stopping incidents. Then, in the second instance, they were measured specifically during rush hour around 7:00 am and 5:30 pm when the train may stop due to congestion. This second instance was particularly challenging to compile, since we only targeted certain routes where the landmark references can be observed (as outlined in Section 7.2) during these rush hours. Unfortunately, we only managed to record 6 out of 11 lines for this test instance, where stopping incidents happened. The Waterloo & City, Hammersmith & City, Circle, Metropolitan, and Bakerloo lines ran smoothly during all of our rush-hour test trips over a 4 month period. In total, we observed 56 stopping incidents across 4 test phones. Additionally, there were 48 landmark references on these routes, which gives us a total of 104 test samples for the rush-hour instance.

### 7.5. Temporal and Spatial Variation of the Accelerometer Trace across the Underground Network

The purpose of this experiment is to verify whether the accelerometer produces similar readings on the same route at any time but distinguishable readings on different routes.

Firstly, we compute the DDTW score of every single co-located segment on all 11 lines from the training database to the same segment from each of the 4 test devices. In total, we have 2960 such pairs of segments to assess.

**Figure 13:**
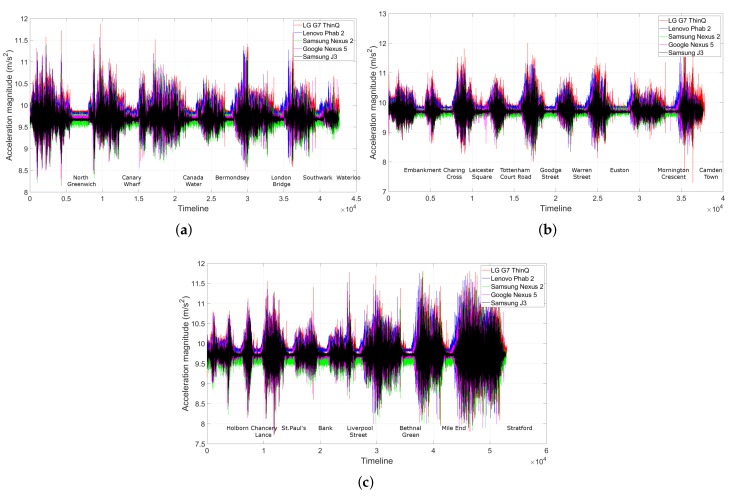
Visual comparison of the shape of the accelerometer traces on the three busiest routes recorded by 5 different phones in various placements and at different times: Their shapes look remarkably similar. The small shift amongst the traces was caused by different sensor’s sensitivities. We do not show the other lines as we observe a similar result. (**a**) Jubilee line; (**b**) Northern line; and (**c**) Central line.

Visually speaking, the shape of the accelerometer traces was remarkably similar (see Figure 13). The small differences were caused by different phones’ sensitivity and by the occasional human gestures, which will be addressed by DDTW. Here, we present just a few examples of the traces on the three most crowded routes, for visual comparison.

**Figure 14:**
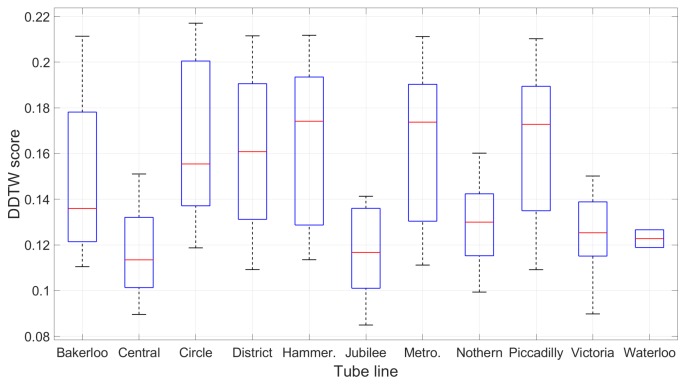
The distribution of the DDTW score for every possible 1-stop co-located trip on each line between the training database and each of the 4 test devices.

Statistically speaking, the maximum DDTW difference score across all lines is just under 0.22 and the mean difference is 0.14, which justifies our empirical selection of the upper-bound threshold of 0.3 to validate the matching pair (as in Section 4.7). Figure 14 details the distribution of the DDTW scores on each line.

Secondly, to put these numbers into perspective, we will compute the DDTW scores between each segment on the training database with other non-co-located segments on the same database and plot them against the scores of the co-located ones computed above. However, because of the sheer number of possible non-co-located pairs (up to 273,430), we randomly picked 3000 pairs spread out across the 11 lines for visualisation purpose. Intuitively, these non-co-located DDTW scores are expected to be much bigger than their co-located counterparts.

**Figure 15:**
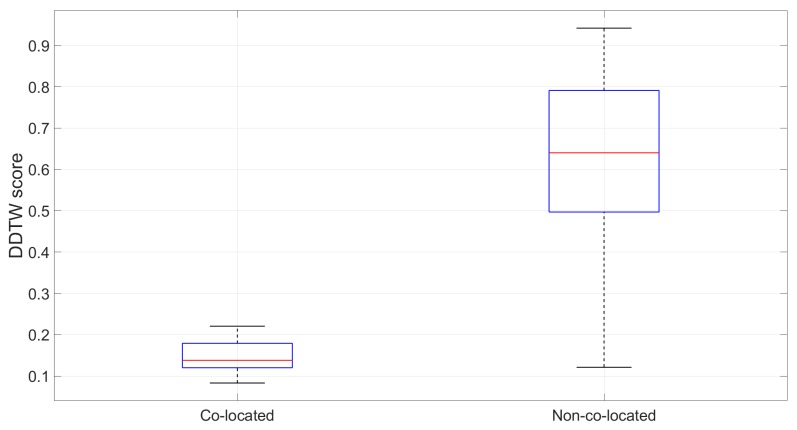
Comparison of the DDTW score between 1-stop co-located and non-co-located trips in the training database.

The result demonstrates a clear difference between an average 0.63 score for the non-co-located routes to that of 0.14 for the co-located ones, and the majority of non-co-located routes scored over 0.5 while co-located ones are always below 0.3 (see Figure 15). Nevertheless, there were some overlapped scores between them, which indicates there are non-co-located 1-stop routes with similar accelerometer measures. The next section will examine whether these routes will cause any wrong line and station estimation and whether they are still a problem for longer multi-stop routes.

### 7.6. Rail Line and Station Estimation Results

The purpose of this experiment is to assess how well our system estimates the correct line and the correct starting station given different accelerometer data’s lengths.

Using the normal instance test set, we start with a 1-stop route and, then, expand them to a 2-stop route, 3-stop route, etc. In short, we have 2960 such 1-stop test routes, 2880 2-stop test routes, 2800 3-stop test routes, and 2720 4-stop test routes. As we will see from the results, it is not worth experimenting sub-one-stop, as we would end up with many wrong estimations because of insufficient sensor data and the similarity amongst routes. Every single test route on all 4 test phones will be compared to the training routes in our database, with the closely matched route chosen as the estimation. It is worth reminding that, for every route with 1 or more stops, the time length heuristic will automatically engage to limit which training routes are compared.

**Figure 16:**
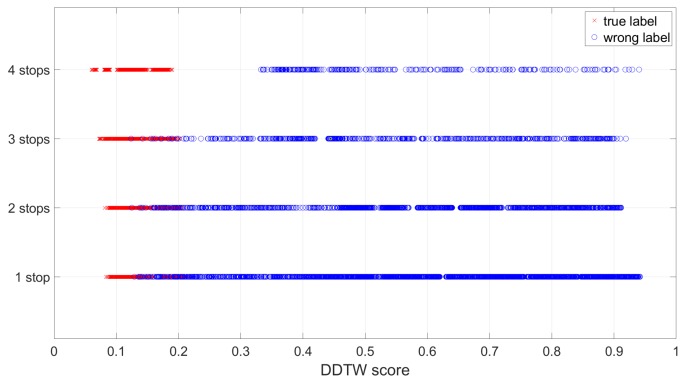
The distribution of the DDTW scores between all the test routes and the training routes: The longer the route is, the clearer the separation between the true co-located routes and the false non-co-located routes is.

We first plot the DDTW scores between all of the above test routes and the training routes to inspect their distribution. Ideally, for a test route to be correctly estimated, the score between itself and the co-located true training route should be the smallest one. It is interesting to observe that the longer the route is, the clearer the separation between the scores of the co-located pairs and the non-co-located pairs is (see Figure 16). There were many overlapped scores for shorter routes, which indicates that there is a chance a short test route may be misclassified, to be reported next.

By comparing the true line and station label of the training routes with the smallest DDTW score to those of the test routes, the results indicated that our approach can achieve 100% accuracy for both line and station estimation anywhere on the London underground when the the passenger has travelled through at least 4 stops (see Table 5). At 3 stops, all of the lines achieved perfect accuracy except the Circle and District lines, since these 2 lines share most of the routes and the stations as well as both use the same rolling stock. To understand the performance on individual lines, we present the confusion matrices with the detailed breakdown of precisely how each test route is classified on each line (see Table 6).

To put these numbers into perspective, we compare them to the real-life average journey length that most London passengers actually spend. Although we do not have the true number of stops as the trip length varies across 11 lines, the official report from Transport for London indicates the average customer’s trip length of the past 15 years was 27.73 min per day (http://content.tfl.gov.uk/travel-in-london-report-8.pdf, p. 79—last accessed in June 2019). Given that the majority of stops was 2–3 min, it is safe to assume that most users would have travelled through at least 10 stations per day.

### 7.7. Speed and Positioning Estimation Results

The purpose of this experiment is to test how close our system’s estimated speed and position are to the actual ones. We focus entirely on the rush-hour test instance, where trains stop midway and the PCA speed estimation engine kicks in. Our readers may wonder why we did not engage the inertial engine right at the beginning of the journey (i.e., completely ignoring the training database from the start and just adding up the acceleration as the train moves), which would give us a whole lot of test cases rather than just 104 test samples in the rush-hour instance while also spares us the hassle of generating a separate test instance. The rationale is that, although this is simple to execute, it is not a fair, realistic experiment because London underground trains do not stop immediately after departing from a station (i.e., they would rather wait at the platform if there is a nearby congestion). Realistically, they would stop somewhere in between the 2 stations or, more often, near the destination station while waiting for the next train to depart. This setting is very important to restrict the accelerometer drifting over time, as the travelling route is rather short.

**Figure 17:**
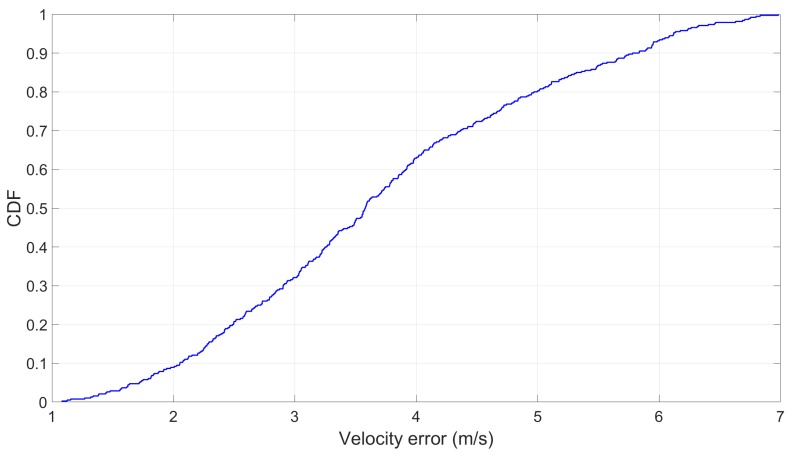
The velocity error with the stopping references: This is what the estimated speed is when the train has actually stopped.

The results suggested that none of the estimated train speeds were close to zero by the time the train had fully stopped for all 381 stopping references. On average, the difference was about 3.7 m per s (see Figure 17). This is the consequence of the accumulated velocity estimation errors.

**Figure 18:**
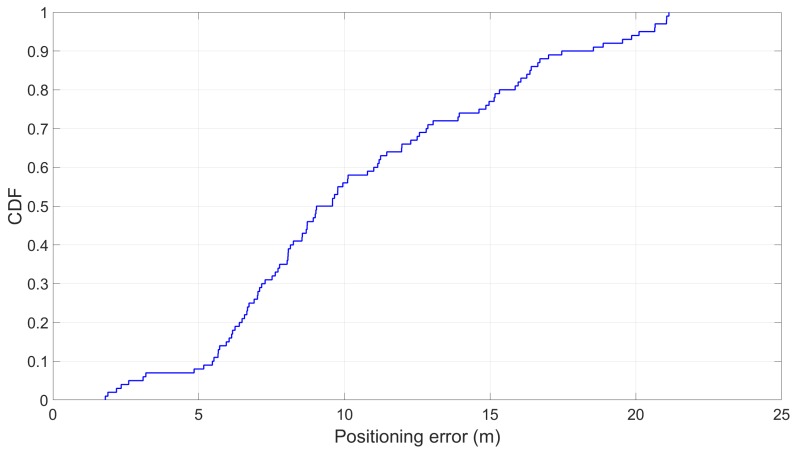
The positioning error using open space landmarks.

Subsequently, the estimated position which is deduced from the train velocity is also affected. The results suggested that the positioning error (i.e., the difference between the actual travelled distance and the estimated one) can be as high as 21 m (see Figure 18). To put this number into perspective, the average length of all the shortest stops across all 11 lines is 590 m.

## 8. Related Work

This section overviews some of the most recent work in the underground transport tracking area, emphasising how we improved on their work.

Transport for London have trialled two solutions to track underground passengers. The first one used Oyster cards, which are used to enter the gates. By recording the entrance and exit stations, they have a rough idea of the passengers’ routes. Nevertheless, since there are several paths to travel between two stations, it is unclear which routes are actually used by the passengers with this approach. The second solution which was revealed in late 2017, used the Wi-Fi Access Points (APs) to record the passengers’ presence at the platform level, based on their phone’s MAC address. The disadvantage of this approach is that there are no Wi-Fi APs installed anywhere in the London underground tunnels but only at the station platforms, at this time of writing. Hence, passengers cannot track their positions in between stations. This approach also requires a central server to collect and process passengers’ data, which may not scale with huge number of users, and has certain privacy concerns. We offer a decentralised approach, where the users have full control of their information.

Due to the lack of GPS underground, other researchers have also turned their attention to smartphones sensors. Lee et al., Higuchi et al., and Nguyen et al., used the magnetometer to measure the magnetic disturbance underground [20,21,22]. The challenge of using the magnetic field is that it is disturbed not only by the tunnel’s infrastructure but also by the train’s on-board electricity, which, despite being spatially unique, is not temporally distinctive. Hence, magnetism is limited to co-location purposes only. Stockx et al. also used the accelerometer to estimate the passenger’s position [23]. However, while they used the Android’s linear accelerometer, a virtual sensor that filters out gravity based on the magnetometer, which is noisy underground, we improved on their work by using PCA to estimate the gravity vector and worked out the true train movements with only the accelerometer measures. Additionally, we proposed an accelerometer database while they convert the acceleration directly into positions. Hyuga et al. and Watanabe et al. proposed to use the barometer to estimate the state motion of the passenger and the latest stop based on the elevation of the stations [24,25]. Lee et al. [26,27] proposed to use the Wi-Fi Access Points to track the station stops in a similar manner to the TfL approach discussed above. Gu et al., Hur et al., Maier et al., and Mongia et al. used a combination of smartphone sensors to classify passenger activities on underground transports [28,29,30,31,32]. We improved on their work by using the passengers’ activities to identify the beginning and ending of a train journey for route matching.

## 9. Conclusions and Further Work

We have presented an approach to track the passengers’ position on the London underground tubes. The novelty of our work is the use of an accelerometer training database describing the unique shape of the entire underground network including the bumps, vibrations, and curves in the language of the sensor measures. The passenger’s position can then be inferred through pattern matching. We exploit the observation that London tubes are self-driving autonomous vehicles travelling on fixed rail lines, which allows us to capture such accelerations and decelerations. We have discussed the challenging situation where the train may stop mid-route due to congestion, which invalidates the use of our proposed training database, by proposing an inertial tracking approach with PCA. The empirical results performed across all 11 lines of the London underground demonstrated that our system can achieve 100% line and station estimation accuracy when the passenger has travelled through at least 4 continuous stops. With only PCA-based positioning estimation, we achieved up to 18 m accuracy, 90% of the time.

In the future, if the train carriage mechanics are upgraded, part of the training database where that train operates may need updating for the most accurate positioning results. However, the shape of the London tunnel should remain largely the same due to its 150-year-old age, which is reflective in the accelerometer traces (i.e., a train trip may now be shorter, but the peaks and bumps on the trace should be similar). With Derivative Dynamic Time Warping, we perform trace matching by exposing the true shape of the tunnel rather than comparing the exact accelerometer values.

One of the potential expansions of our work is the incorporation of other sensors with reproducible measures over the same journey to improve the uniqueness of each position and, hence, to result in better route-matching accuracy. A second line of thought is that we move away from localisation to co-localisation by detecting when two passengers are nearby. This category has great use cases for epidemic tracking and disease monitoring, which do not require the user’s position.

## Figures and Tables

**Table 1:** An overview of 11 London underground lines sorted in alphabetical order that were surveyed in the training database.

Line Name	Length (km)	Stations (km)	Shortest Stop	Longest Stop (km)	Train Stock
Bakerloo	23.2	25	0.37	1.74	1972
Central	74	49	0.4	3.34	1992
Circle	27.2	36	0.31	1.85	S7
District	64	60	0.31	2.37	S7
Hammersmith & City	25.5	29	0.51	2.29	S7
Jubilee	36.2	27	0.44	2.85	1996
Metropolitan	66.7	34	0.51	11.63	S8
Northern	58	50	0.27	2.46	1995
Piccadilly	71	53	0.25	4.42	1973
Victoria	21	16	0.74	3.15	2009
Waterloo & City	2.5	2	2.37	2.37	1992

**Table 2:** Summary of the open-air landmarks recorded in the test data.

Line	Landmarks	Comments
Bakerloo	3	by Willesden Junction, Queen’s Park, Wembley Central stations
Central	3	by White City, Leyton, Newbury Park stations
Circle	8	by Edgware Road, Bayswater, Notting Hill Gate, High Street Kensington, South Kensington, Sloane Square, Farringdon, Barbican stations
District	5	by Ravenscourt Park, South Kensington, Sloane Square, Whitechapel, Bow Road stations
Hammersmith & City	5	by Edgware Road, Farringdon, Barbican, Whitechapel, Bow Road stations
Jubilee	2	by Finchley Road, Canning Town stations
Metropolitan	3	by Finchley Road, Farringdon, Barbican stations
Northern	4	by Hendon Central, Hampstead, East Finchley, Morden stations
Piccadily	5	by Hounslow West, Hammersmith, Baron Courts, Bounds Green, Southgate stations.
Victoria	0	the entire tunnel is underground
Waterloo & City	0	the entire tunnel is underground

**Table 3:** Overview of the smartphones and the accelerometers used in the experiments: The LG G7 ThinQ was used for training, while the other four were used for testing.

Phone Model	Accelerometer Vendor	Chip Revision	Year Made	Sampling Rate
LG G7 ThinQ	Invensense	32	2018	50 Hz
Samsung J3	Bosch	1	2017	10 Hz
Lenovo Phab 2	Bosch	1,173,700	2016	20 Hz
Google Nexus 5	Invensense	1	2013	30 Hz
Samsung Nexus 2	Invensense	1	2011	40 Hz

**Table 4:** Summary of the two test instances generated by 4 test devices: The first instance covers normal train routines used for the temporal, spatial, and pattern matching validation. The second instance covers rush-hour traces, used for estimating speed validation. The term “test samples” below refers to the route between two consecutive stations in the normal instance and to the stopping landmark references in the rush-hour instance.

	Test Samples	Stations	Lines
Normal instance	2960	381	11
Rush-hour instance	104	28	6

**Table 5:** The line and station estimation accuracy: Overall, the longer the passenger travels, the more accurate the estimation is. Absolute 100% accuracy was achieved on both line and station estimation when we have at least 4 stations’ length of accelerometer data.

	Line Estimation				Station Estimation			
	1 Stop	2 Stops	3 Stops	4 Stops	1 Stop	2 Stops	3 Stops	4 Stops
Bakerloo	41.67%	73.9%	100%	100%	35.42%	67.39%	100%	100%
Central	53.13%	74.4%	100%	100%	45.83%	70.21%	100%	100%
Circle	32.86%	60.3%	91.43%	100%	15.71%	57.35%	84.29%	100%
District	48.3%	62.1%	93.22%	100%	33.9%	56.9%	90.68%	100%
Hammersmith & City	19.64%	46.3%	100%	100%	16.1%	44.4%	100%	100%
Jubilee	46.15%	70%	100%	100%	40.38%	62%	100%	100%
Metropolitan	21.2%	43.8%	100%	100%	16.7%	40.6%	100%	100%
Northern	63.27%	79.2%	100%	100%	54.1%	74%	100%	100%
Piccadilly	44.23%	65.7%	100%	100%	34.62%	62.75%	100%	100%
Victoria	53.3%	85.7%	100%	100%	46.67%	78.6%	100%	100%
Waterloo & City	100%	100%	100%	100%	100%	100%	100%	100%

**Table 6:** The confusion matrix for line and station estimation: The columns represent the true classes. The rows represent our system’s predictions. The diagonal number which is highlighted in bold, reports the correct line classifications for each class, with the number inside the bracket as the correct station estimation. We do not provide the table for 3 stops since only the District and Circle lines were misclassified in this case. Note: Hammer. is short for the Hammersmith & City line and Metro. is short for the Metropolitan line due to space limit.

						1 stop						
	**Bakerloo**	**Central**	**Circle**	**District**	**Hammer.**	**Jubilee**	**Metro.**	**Northern**	**Piccadilly**	**Victoria**	**Waterloo**	**Total**
Bakerloo	**80 (68)**	8	16	40	16	12	8	4	8	0	0	192
Central	0	**204 (176)**	0	56	4	28	8	48	24	12	0	384
Circle	12	4	**92 (44)**	76	20	0	44	4	28	0	0	280
District	52	16	68	**228 (160)**	44	4	52	0	8	0	0	472
Hammer.	12	0	32	56	**44 (36)**	0	32	8	36	4	0	224
Jubilee	4	36	0	16	0	**96 (84)**	0	52	0	4	0	208
Metro.	8	0	52	64	36	0	**56 (44)**	0	48	0	0	264
Northern	0	36	12	20	8	48	0	**248 (212)**	12	8	0	392
Piccadilly	8	16	52	40	52	0	60	4	**184 (144)**	0	0	416
Victoria	0	8	0	0	8	16	0	24	0	**64 (56)**	0	120
Waterloo	0	0	0	0	0	0	0	0	0	0	**8 (8)**	8
Total	176	328	324	596	232	204	260	392	348	92	8	2960
						**2 stops**						
	**Bakerloo**	**Central**	**Circle**	**District**	**Hammer.**	**Jubilee**	**Metro.**	**Northern**	**Piccadilly**	**Victoria**	**Waterloo**	**Total**
Bakerloo	**136 (124)**	4	0	24	4	12	0	0	4	0	0	184
Central	0	**280 (264)**	0	32	0	16	4	24	12	8	0	376
Circle	0	0	**164 (156)**	60	8	0	24	0	16	0	0	272
District	32	16	52	**288 (264)**	36	0	36	0	4	0	0	464
Hammer.	0	0	16	48	**100 (96)**	0	20	4	28	0	0	216
Jubilee	0	20	0	8	0	**140 (124)**	0	28	0	4	0	200
Metro.	0	0	36	48	28	0	**112 (104)**	0	32	0	0	256
Northern	0	16	8	12	8	24	0	**304 (284)**	4	8	0	384
Piccadilly	0	0	36	28	32	0	44	0	**268 (256)**	0	0	408
Victoria	0	0	0	0	0	4	0	12	0	**96 (88)**	0	112
Waterloo	0	0	0	0	0	0	0	0	0	0	**8 (8)**	8
Total	168	336	312	548	216	196	240	372	368	116	8	2880
